# Hematopoietic Stem and Progenitor Cells Establish Dynamic Podosome‐Like Structures to Interact with Fibronectin

**DOI:** 10.1002/adbi.70159

**Published:** 2026-09-27

**Authors:** Miriam Greve, Peter Schertl, Frank Schaarschmidt, Constantin von Kaisenberg, Cornelius Miller, Susanna Spindler, Sophia Rudorf, Cornelia Lee‐Thedieck

**Affiliations:** ^1^ Institute of Cell Biology and Biophysics Leibniz University Hannover Hannover Germany; ^2^ Hannover Medical School Department for Obstetrics Gynecology and Reproductive Medicine Hannover Germany

**Keywords:** adhesion, bone marrow niche, fibronectin, hematopoietic stem cells, podosomes, Proline‐Rich Tyrosine Kinase 2 (PYK2)

## Abstract

To keep their stem cell properties, hematopoietic stem and progenitor cells (HSPCs) need to interact with their stem cell niche in the bone marrow. Yet, the way in which HSPCs anchor to this specific microenvironment composed of niche cells and extracellular matrix (ECM) is not well understood. Here, the adhesion structures established by HSPCs upon contact with the ECM component fibronectin are investigated with regard to their appearance, maturity state, and composition. HSPCs adhere in a dynamic manner and display varying morphologies, with most cells displaying a round shape and the establishment of many podosome‐like structures (PLS). The PLS consist of F‐actin puncta, co‐localized with phosphorylated PYK2, vinculin, and paxillin. These proteins assemble towards the periphery of the structure, and this pattern is more pronounced in cells with a round shape and higher PLS numbers. We also find strong indications that cells with a round shape or displaying higher levels of PLS are less differentiated and less likely to be of myeloid lineage. The study highlights the dynamic adhesion behavior of HSPCs and points to PLS as structures of interest in the interaction between these cells and their niche. Podosome‐like structures are found in the majority of hematopoietic stem and progenitor cells. These structures are more maturely established in round cells, and cells establishing the structures in higher quantities are more naïve.

## Graphical abstract

Hematopoietic stem and progenitor cell (HSPC) adhesion to the extracellular matrix (ECM) is crucial for their functionality. HSPC adhesion to the ECM component fibronectin is dynamic and goes along with various cell morphologies and podosome‐like structures (PLS). Assessing adhesive structures together with cell surface markers reveals that cell morphologies and PLS abundance correlate with HSPC lineage and differentiation.

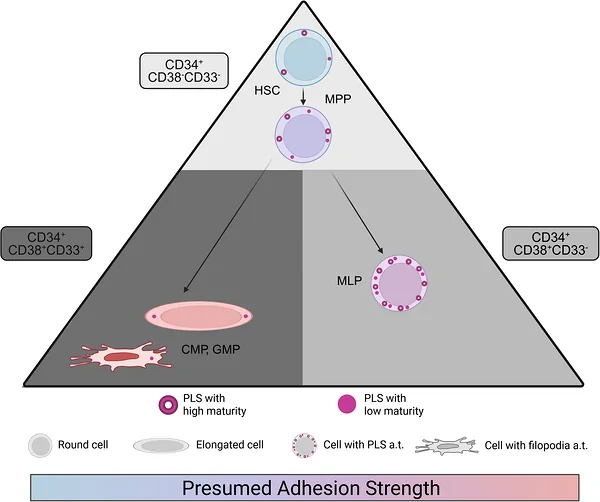

## 1 Introduction

Throughout life, blood cells need to be constantly replaced due to their limited lifespan. This task is fulfilled by hematopoietic stem cells (HSCs). These cells are defined by their abilities to self‐renew and to give rise to all cells of the hematopoietic system through differentiation. HSCs are only able to maintain their stemness if they reside within their specific stem cell niches in the bone marrow (BM). These specialized microenvironments near the endosteum and arterioles or close to sinusoids supply hematopoietic stem and progenitor cells (HSPCs) with the signals they need to regulate self‐renewal and differentiation according to the needs of the organism [1, 2]. The BM niche provides these signals via a network of niche cells, soluble factors such as cytokines or chemokines, and the extracellular matrix (ECM). The interplay of all these components, together with biophysical cues within the BM niche, provides the only known conditions in which HSPCs can maintain their stemness, as they start to differentiate quickly once they exit the BM niche [2, 3, 4]. Given the importance of the cell microenvironment in maintaining stemness, a multitude of bone marrow niche models have been developed to expand and research HSPCs ex vivo [5]. The replication of the ECM in composition and structure is a focus of these ongoing efforts, as it provides important anchorage points for HSPCs to adhere to and thus remain in their niche in vivo. A ubiquitously present adhesion substrate in the ECM of the BM is fibronectin (FN) [6]. The glycoprotein provides a multitude of binding sites for a variety of adhesion receptors, mainly integrins [7]. Most importantly to the study presented here, FN offers binding sites to integrins α4β1, α5β1 and αvβ3 [7, 8], which have been shown to sustain the homeostasis of long‐term HSCs [9, 10, 11]. The prevalence of FN in HSPC attachment is so significant that it is frequently used to study the adhesion behavior of these cells [12].

Integrin binding leads to the establishment of adhesion structures by cells. Well‐known adhesion structures are focal adhesions, which are established by, e.g., fibroblasts. They consist of dense plaques of F‐actin with a width of 2–6 µm [13]. An early event in focal adhesion establishment is the clustering of integrins upon ligand binding, an event that takes place in filopodia that protrude beyond the cell edge [14, 15]. Focal adhesions are sites of force transmission, and the interplay of talin, vinculin, and paxillin within the adhesion sites, facilitating the bi‐directional mechanotransduction between cell and matrix, is necessary for maturation of focal adhesions [13, 15]. Other adhesion structures that are established upon integrin binding are invadosomes. Invadosomes are structures that are closely related to matrix degradation. They consist of an actin‐rich core, surrounded by a ring of adhesive molecules, e.g., vinculin and paxillin [16]. Invadosomes are categorized as podosomes if they are established by somatic cells, e.g., dendritic cells, or as invadopodia if the structures are established by cancer cells. An early event in podosome formation is the phosphorylation of the proline‐rich tyrosine kinase 2 (PYK2), a relative of the focal adhesion kinase (FAK), which localizes to the adhesive ring of the structures [17]. The recruitment of vinculin and paxillin to the ring structure marks the maturation of the structures [16, 18]. Focal adhesions and invadosomes share much of the same molecular machinery, whereas they differ greatly in architecture and half‐life. While podosomes usually persist in the range of minutes, invadopodia and focal adhesions can be stably present in the range of hours [13].

A number of cell adhesion molecules have been shown to have an impact on HSPC maintenance, including integrins [3]. Integrin engagement in HSPCs is mediated via PYK2, which is produced in high amounts by HSPCs at the expense of its relative, FAK, and has been shown to lead to quiescence and recruitment of paxillin in these cells [13, 19, 20, 21]. Furthermore, vinculin has been shown to be necessary for HSPC repopulation after transplantation [22]. Although all these cell adhesion molecules are involved in HSPC maintenance and are present in both focal adhesions and invadosomes, none of these adhesion structures have been shown to be established by HSPCs in a fully mature state so far. Rademakers et al. [23] showed the establishment of podosome‐like structures (PLS) in HSPCs upon stimulation of the cells with a phorbol ester. These structures were discussed by the authors as being involved in transcellular migration during homing of the cells. Whether these structures are also involved in retention of the HSPC in the niche by integrin‐mediated adhesion to the ECM, or if HSPCs also establish these structures without stimulation, is still unknown.

The aim of the here presented study is to reveal what kind of adhesion structures are established by HSPCs in contact with FN, and to analyze them in regard to their appearance, maturity state, and molecular composition. For this purpose, human primary HSPCs adherent to FN were analyzed via super‐resolution microscopy. In this way, PLS could be identified as a primary adhesion structure mediating the dynamic adhesion of HSPCs to FN. Adhesion markers were visualized in the structures to gain insight into the spatial organization of PLS. Furthermore, analysis of adhesive structures and cell morphology in dependence on early hematopoietic differentiation indicates that cell adhesion and the established adhesive structures are modulated in early differentiation. Given the importance of adhesion mechanisms in the context of HSPC maintenance, these findings could provide new insights into HSPC‐niche interactions and how to exploit these mechanisms to expand HSPCs ex vivo.

## 2 Results

### 2.1 Dynamic Adhesion Behavior in HSPCs Results in Different Presentation of the F‐actin Cytoskeleton

The adhesion behavior of CD34^+^ HSPCs isolated from umbilical cord blood (UCB) on FN was assessed via live cell imaging initially. The cells displayed a highly dynamic adhesion behavior, where cells attached, detached, and showed rapid migration or retention on the substrate. Some of these migrating cells were able to span a distance twice their own size within a 10 min period, and almost 100 µm in less than 60 min (Figure 1A). Once cells were fixed, labelled with fluorophore‐coupled phalloidin and imaged via confocal laser scanning microscopy (cLSM), only the cell fraction displaying adhesion forces strong enough to withstand the washing and labelling procedure was analyzed. Yet the dynamic adhesion behavior observed in live cell imaging also translated to this cell population via various presentations of the F‐actin cytoskeleton, with cells displaying round or elongated cell shapes, establishing F‐actin structures such as arcs, PLS and/or filopodia (Figure 1B). PLS appear as spots of F‐actin with high fluorescence intensity and a diameter below 1 µm, filopodia protrude beyond the cell edge, and F‐actin arcs are visible as bundles of stress fibers in the cell periphery. This heterogeneity of morphologies and the diversity of F‐actin structures within them, as well as the dynamic adhesion behavior, prompted the establishment of a grouping process for cells to get a clearer understanding of the structures, as a rapid interchange between morphologies and structure assembly/disassembly was indicated by these results. Basically, the approach aimed to define the structures in their most pronounced state. Thus, the images of adherent cells were submitted to a categorization process to group them according to cell shape and established F‐actin structures. Briefly, cells were categorized as round or elongated via the aspect ratio (AR). Large, polygonal cells displaying actin arcs, identified by cell area, were categorized as arc cells. Each analyzed cell was assigned to one of these three categories. Additionally, to determine cells displaying PLS and filopodia, values acquired with the ImageJ plugins “Poji” and “Filoquant” were used, respectively. A threshold for these values was determined by selecting cells that showed an abundance of the aforementioned structures and determining the median of chosen values between these cells and the rest of the population (Table 1; Figure S1). Parameters of the plugins were kept constant to ensure comparability. In this way, cells were assigned the PLS and filopodia labels, which, due to using the median as a threshold, did not indicate whether the structures were present at all, but rather that the structures were present above the set threshold. In the resulting distribution of morphologies and adhesion structures across adherent HSPCs (Figure 1C), the majority of cells were round with PLS or filopodia numbers below threshold (b.t.) (44.5 ± 14.8%), followed by the group of round cells presenting PLS above threshold (a.t.) that made up 24.7 ± 14.7% of the cells. Cells with a round cell shape displaying filopodia or PLS and filopodia a.t. were the next most common, with 9.7 ± 2.8% and 7.0 ± 1,4% respectively (mean and standard deviation of percentages of cells of respective category from *N* = 3 independent donors). Elongated cell shapes were less frequent in adherent HSPCs; each category, in combination with and without F‐actin structures a.t., made up less than 5% of the total cell population. Lastly, cells that established F‐actin arcs were rare, with less than 1%, and filopodia a.t. only appeared together with PLS a.t. in these cells. In subsequent analyses of cell distributions, this rare population did not account for a significant portion of the data and was therefore disregarded. The amount of PLS and filopodia as F‐actin structures a.t. correlated with cell shapes. Only half of the round cells displayed PLS and/or filopodia a.t., with cells showing PLS a.t. being more frequent (∼29%) than cells with filopodia a.t. (∼11%) or both structures a.t. (∼8%). In contrast, among elongated cells, the majority (∼71%) showed these distinct adhesive F‐actin structures a.t., mostly displaying filopodia along with PLS a.t. or filopodia alone a.t. (∼30% and 28%, respectively). Only 14% of the elongated cells showed PLS a.t. with filopodia b.t. (Figure 1D). Accordingly, when assessing vice versa, the cell shape distribution among cells with PLS and filopodia a.t., an inverted relationship was observed, where 68% of cells with filopodia a.t. are elongated, while 84% of cells with PLS a.t. showed a round shape (Figure S2).

**FIGURE 1:**
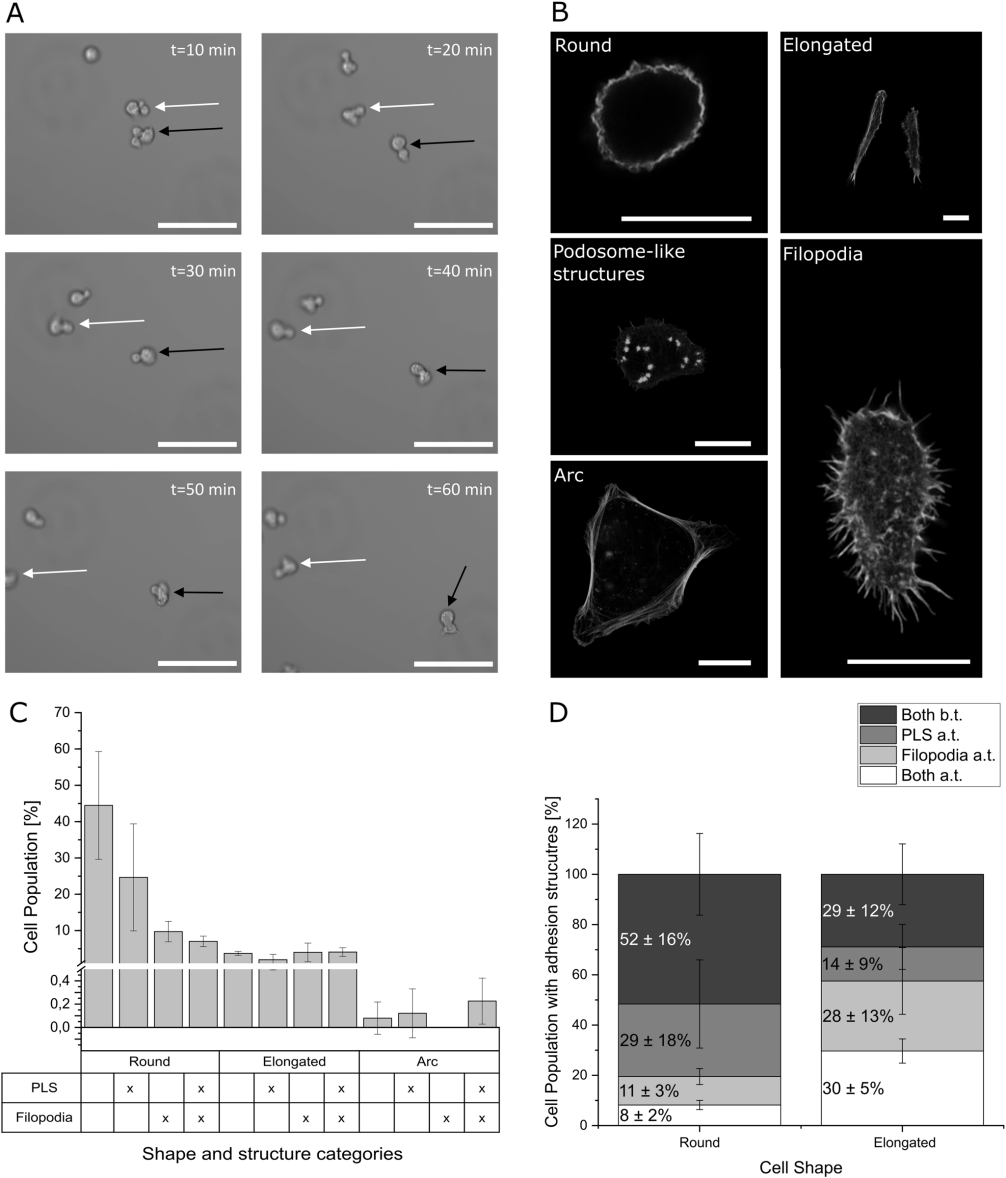
HSPCs in contact for 1 h with FN show dynamic adhesion behavior, which is reflected in a variety of cell shapes and adhesive structures. (A) Live cell imaging of HSPCs isolated from UCB in contact with FN. Cells were allowed to adhere to FN for 1 h in total; images at 10 min intervals are shown. While some cells remain on the substrate throughout the experimental procedure (e.g., cell marked with a black arrow), other cells show high migratory behavior spanning a 100 µm range in less than 60 min (e.g., cell marked with a white arrow). Scale bar = 50 µm; (B) Cells were let to adhere to FN for 1 h and subsequently fixed, fluorescently stained for F‐actin (light grey), and submitted to super‐resolution confocal microscopy. The cytoskeleton of cells presented heterogeneously, with cells taking on round or elongated shapes, establishing F‐actin arcs in the cell periphery or displaying PLS or filopodia. Scale bar = 10 µm; (C) The population sizes of cells with distinct shapes and adhesive structures reveal that the majority of HSPCs have a round shape. A large population of cells with a round shape establishes amounts of PLS above the set threshold. Cells of elongated shape only make up ∼ 14% of the entire cell population. HSPCs with F‐actin arcs are rarely observed, collectively making up <1% of the entire population. Depicted are the mean (± SD) population sizes of respective categories of *N* = 3 independent UCB donors. The table shows cell shape and structure category, with (x) indicating PLS/filopodia presence above the set threshold; (D) Percentage of cells above (a.t.) and below (b.t.) the threshold of adhesive structures in round or elongated HSPCs. More than half of the cells with a round cell shape do not show a considerable number of structures; however, round cells that do display structures mostly show PLS (∼29%). In elongated cells, ∼71% show large numbers of structures with filopodia or filopodia together with PLS structures being predominantly present. Depicted are the mean (± SD) population sizes of respective categories of *N* = 3 independent UCB donors.

**TABLE 1:** Threshold values for morphology assignment in adherent HSPCs.

Morphology descriptor	Threshold value
Arc	Cell Area ≥ 346 µm^2^
Elongated	AR ≥ 2
Round	AR < 2
Filopodia	Total filopodia number ≥ 17 and mean filopodia length ≥ 0.4 µm
PLS	Total podosome number ≥ 15 and/or podosome density ≥ 0.08 PLS µm^−2^

### 2.2 Podosome‐Like Structures in HSPCs Show Multiple Assembly States

Since cells with PLS numbers a.t. accounted for a large percentage of adherent HSPCs, the cells were labelled for proteins that serve as markers for native and mature podosomes to gain further insight into the maturity state and similarity of these structures to podosomes. Phosphorylated PYK2 (pPYK2) was chosen as a marker of early structure establishment, while vinculin and paxillin were used to label more mature structures. In HSPCs, F‐actin puncta were clearly colocalized with pPYK2 throughout all cells that displayed these structures (Figure 2A). Similarly, in most cells, vinculin and paxillin were found to be accumulated at the puncta (Figure 2A). However, it could also be observed in some cells that the markers formed a ring around F‐actin puncta, as would be expected for mature podosome structures.

**FIGURE 2:**
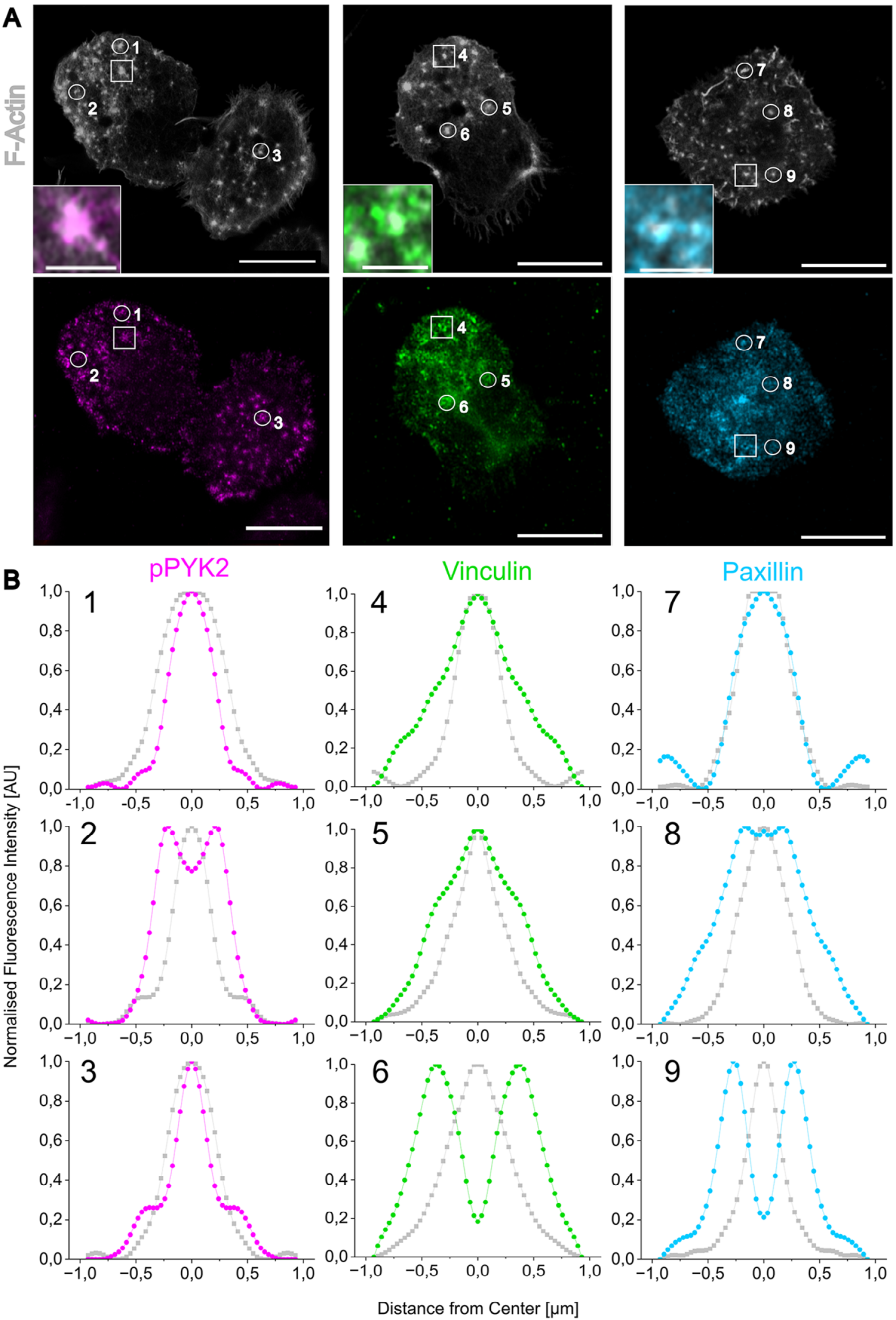
The assembly states of PLS in adherent HSPCs are heterogeneous even at the single‐cell level. HSPCs were isolated from UCB and allowed to adhere to FN for 1 h. (A) IF images of adherent HSPCs acquired by cLSM show the distribution of pPYK2 (magenta), vinculin (green), and paxillin (blue) relative to F‐actin (grey) puncta. Small squares indicate areas of enlarged images of PLS insets in F‐actin images. (Images representative of *n* = 99 cells of PLS category from *N* = 3 UCB donors; Scale bar = 10 µm, Scalebar insets = 2 µm); (B) Radial intensity profiles of individual PLS, labelled with corresponding numbers in subimage (A), show the heterogeneous assembly of the structures even at the single‐cell level. Radial Intensity Profiles show the spatial distribution of pPYK2, vinculin, and paxillin in relation to F‐actin puncta according to the normalized fluorescence intensity in labelled structures. Colors of (A) apply.

In order to define the spatial distribution of pPYK2, vinculin, and paxillin in relation to the F‐actin puncta, the ImageJ plugin “Poji” was used to acquire radial intensity profiles (RIPs). These allow assessment of the distribution of labelled proteins relative to the center of the structure. These analyses revealed that the distribution of the molecules around the F‐actin puncta differed between individual PLS, just as observed before in the images acquired via immunofluorescent microscopy. (Figure 2B). In some structures, a ring formation was clearly visible by a double peak in the RIP, while in others, a simple colocalization of the proteins with the F‐actin puncta was found, as the peaks of the respective RIPs aligned to the center of the structure. These different RIPs were observed even at the single‐cell level, confirming the visual observations of the overall localization of markers. Comparing the RIPs of single PLS with their visual display, a ring formation might be observed in fluorescent images but was not reflected in the profile (e.g., Figure 2, structure 4 and corresponding profile). This phenomenon is the result of the marker proteins distributing in a geometrical but asymmetric manner within the center of the F‐actin structure, resulting in averaging of the signal during the acquisition of the RIP, which is a symmetrical process. This was a further confirmation of the diverse assembly states of PLS even at the single‐cell level. Thus, microscopy images of adhering HSPCs revealed PLS, in which the studied markers differed in their assemblies, most likely representing snapshots of PLS, which were dynamically assembled and disassembled during the dynamic adhesion of HSPCs.

### 2.3 Characteristic Distribution of Podosome‐Markers Within PLS Is More Pronounced in HSPCs with Round Cell Shape and Round Cells with the PLS Label

In the next step, we investigated whether the spatial distribution of the proteins of interest, pPYK2, vinculin, and paxillin, in PLS correlated with different shape and structure categories of the cells previously established. Thus, the averaged RIPs of all cells within the same cell category were analyzed. The averaged RIPs of the proteins of interest showed colocalization with the F‐actin puncta and exceeded their periphery. However, shoulders or double peaks were smoothed out in the averaged profiles, indicating that the majority of PLS was rather immature without clear ring formation of the analyzed proteins around the F‐actin puncta (Figure 3A). To quantify the spatial distribution of the proteins of interest within PLS, the full width at half maximum (FWHM) was calculated from RIPs of individual cells. This confirmed the distribution of proteins of interest with regard to F‐actin observed in the RIPs, where the mean FWHM of pPYK2 was higher than that of F‐actin, and the mean FWHM of vinculin and paxillin, respectively, exceeded that of pPYK2 for all analyzed groups (Figure 3A,B). The analysis of FWHM highlighted two major aspects of PLS in adherent HSPCs: First, the F‐actin puncta did not show significant differences in size throughout all analyzed cells of different shapes and with different adhesive structures. The FWHM of F‐actin came to an average of 365.4 ± 60.0 nm in all populations. Second, the proteins of interest were accumulated closer to the F‐actin puncta in cells with filopodia a.t. This was emphasized by the fact that statistically significant differences in the FWHM of the proteins of interest between groups were only found in combination with the round‐filopodia and the elongated‐filopodia categories, respectively. In detail, the FWHM of pPYK2 was significantly lower in cells of the round‐filopodia category than in cells of the round, elongated, and round‐PLS categories. Further, FWHM values of pPYK2 in the elongated‐filopodia group were significantly lower than in cells that were round or elongated with structure establishment b.t. Lastly, FWHM values of vinculin and paxillin were significantly lower in round cells with filopodia a.t. than in cells of the round and round‐PLS categories, respectively.

**FIGURE 3:**
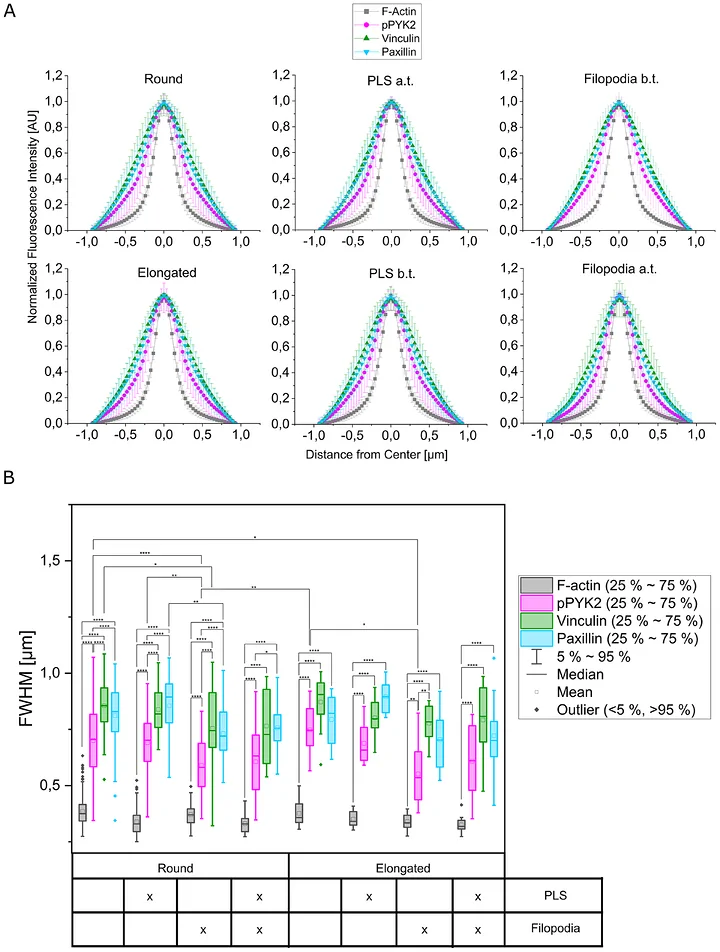
PLS are most mature in HSPCs with round morphology and in round HSPCs with PLS numbers above the threshold. HSPCs were isolated from UCB and allowed to adhere to FN for 1 h. (A) Mean radial normalized intensity profiles ± SD of PLS detected via “Poji” in adherent HSPCs with different morphologies. The total population of adherent HSPCs was separated according to their morphology (round vs elongated, PLS a.t. vs PLS b.t., Filopodia a.t. vs. Filopodia b.t.). The spatial distribution of pPYK2, vinculin, and paxillin in reference to F‐actin within the PLS shows a distribution of the three proteins towards the F‐actin puncta periphery, without formation of a full ring, in all groups. (B) Boxplot depicting the FWHM of radial intensity profiles for F‐actin, pPYK2, Vinculin, and paxillin for PLS from HSPCs within different morphology groups. Statistical analysis with one‐way ANOVA with a Tukey post hoc test. Marked with asterisks are significant differences within the same group or between identical molecules in different groups (^*^: *p* ≤ 0,05; ^**^: *p* ≤ 0,01; ^***^: *p* ≤ 0,001). Radial Intensity Profiles and FWHM data from PLS of *n* = 482 cells from *N* = 3 UCB donors. Table and graph headings refer to cell morphology, (x) indicating PLS/filopodia presence a.t.

Furthermore, while FWHM of all proteins of interest were significantly higher than those of the F‐actin puncta regardless of cell category, the FWHM of vinculin and paxillin were generally larger than those of pPYK2. These differences were most pronounced and statistically significant in round cells and round cells with either the PLS or filopodia a.t., as well as in round cells with both structures a.t. (FWHM of only paxillin significantly larger than the FWHM of pPYK2) and elongated cells with filopodia a.t. (FWHM of only vinculin significantly larger than the FWHM of pPYK2). Taken together, the spatial distribution of pPYK2 towards the PLS’ periphery and of vinculin and paxillin beyond pPYK2 was most pronounced in round cells and round cells with PLS a.t., as indicated by the higher FWHM compared to cells with filopodia a.t. and the significant differences between proteins of interest within these groups.

### 2.4 Round HSPC Establishing PLS Are Less Differentiated

Given the heterogeneity of cell shapes, structure establishment, and PLS assembly states observed in adherent HSPCs, it was assessed whether a correlation between the differentiation state and the shape or observed adhesive structures of adherent cells exists. To find reliable markers of the differentiation state of HSPCs, we assessed the expression of markers across the cell populations originating from HSCs. For this purpose, published data by Pellin et al. [24] were used, which contained full single‐cell transcriptomic data of BM HSPC fully annotated with the respective differentiated sub‐populations. We subjected these data to nearest‐neighbor plotting, enabling the assessment of changes in gene expression along the path of hematopoiesis (Figure S3A). CD38, CD33, and CD45RA were chosen to determine the differentiation state of HSPCs. *CD38* expression was enriched in the most differentiated populations of granulocyte‐monocyte progenitors (GMP), Pre‐B and natural killer cells (PreBNK), and to a slightly lesser extent in megakaryocyte‐erythroid progenitors (MEP) (Figure S3B). *CD33* expression was highest in cells of the GMP population, and cells that show heightened *CD33* expression are found in overlap with the common myeloid progenitor (CMP) population (Figure S3C). *PTPRC*, the gene encoding CD45, was highly expressed throughout the population of BM HSPCs, yet slight enrichment in the HSC, multipotent progenitors (MPP), and multi‐lymphoid progenitors (MLP) fractions was found (Figure S3D). Thus, CD38 starts to emerge in HSCs as they lose stemness and commit to lineage differentiation, and vice versa, cells with low CD38 levels are less mature. CD33 is a marker of myeloid lineage commitment (Figure 4A). CD45RA is a splice variant of CD45, known to be present on MLP cells in the absence of CD38, and to emerge together with CD38 in GMP during myeloid differentiation [25, 26]. High levels of CD45RA in combination with low levels of CD38 reflect naïve cells primed towards lymphoid lineage, while higher levels of both markers indicate further differentiation into myeloid lineage. The expression pattern of PLS marker genes *PTK2B*, *VCL* and *PXN* (genes encoding PYK2, vinculin and paxillin respectively) was homogeneous across the entire HSPC population, with enrichment of *VCL* in the populations of MEP and PreBNK (Figure S3E–G). The involvement of the proteins in the establishment of adhesive structures in HSPC is therefore plausible.

**FIGURE 4:**
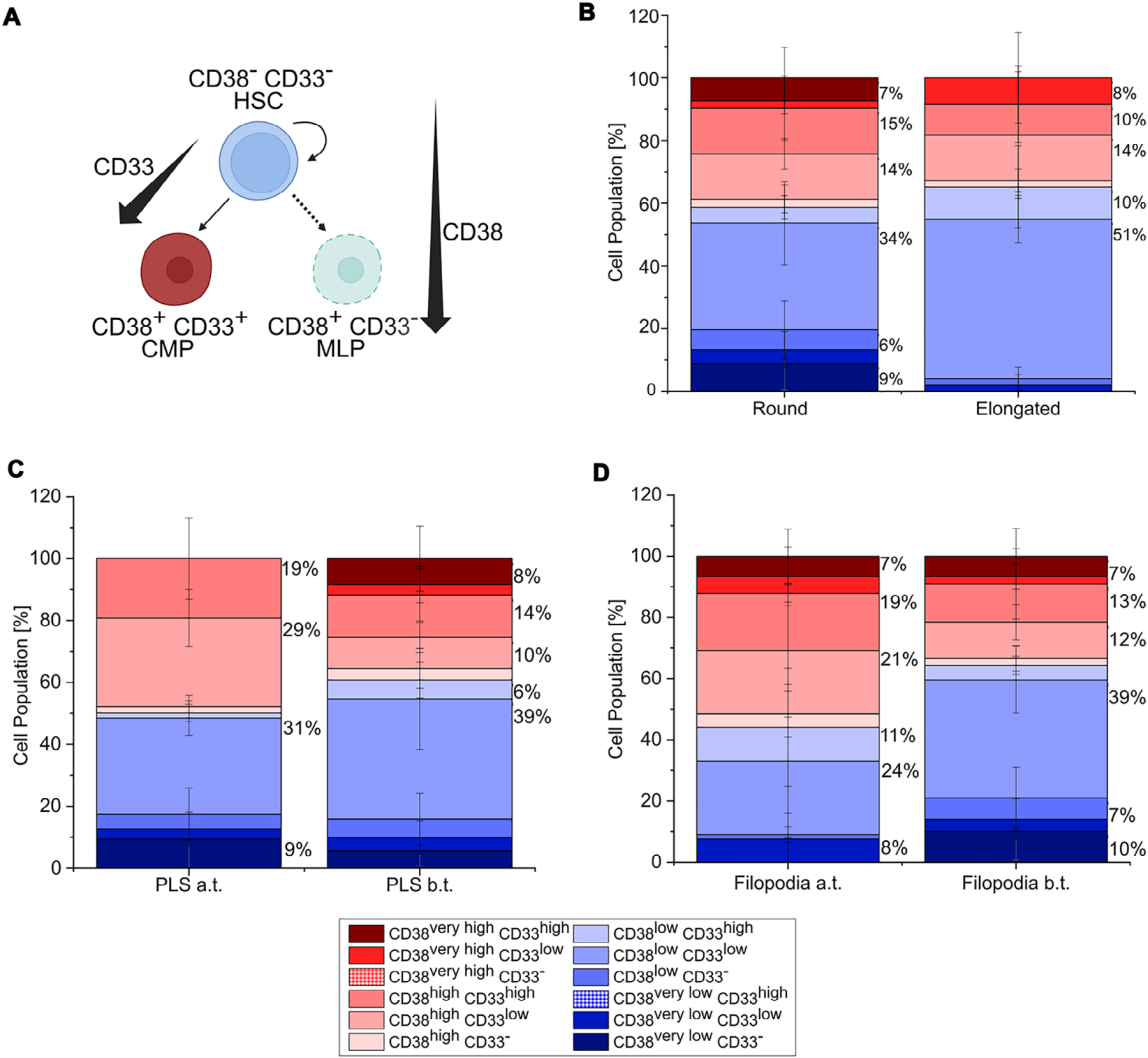
Less differentiated HSPCs are more likely to be round in shape and establish PLS. (A) Proposed progenitor markers to follow HSC differentiation: Naïve HSCs lack both CD38 and CD33; further differentiation is accompanied by loss of self‐renewal marked by CD38 emergence, and myeloid lineage commitment indicated by CD33 expression, as most cells presented positive for CD38 and CD33 and were thus of myeloid lineage. (B–D) HSPCs were isolated from UCB and assessed for progenitor marker production of CD38 and CD33 via Flow Cytometry prior to adhesion. Marker production was assessed via IF microscopy after adhesion to FN for 1 h, and CTCF of the labelled markers was determined. Progenitor marker production levels of cells were grouped according to CTCF values, by transferring population sizes observed in flow cytometry to adhered cells via median CTCF and rule of three. Stacked column graph representing the population sizes of respective marker levels in percent (±SD) of *n* = 249 individual cells from *N* = 3 independent UCB donors. Shown are the population sizes for adherent HSPCs with (B) round vs. elongated morphology, (C) PLS a.t. vs. PLS b.t., (D) filopodia a.t. vs. filopodia b.t.‐ (a.t.) indicating PLS/filopodia presence above the set threshold, (b.t.) indicating PLS/filopodia present below the set threshold, respectively. Mean Percentages >5% are shown next to the respective bar segments.

Initial flow cytometry analysis of the differentiation markers CD33, CD45RA and CD38 on HSPCs from UCB revealed that most of the population was positive for these markers (86.6 ± 18.6% CD33^+^ cells, 94.0 ± 2.6% CD45RA^+^ and 98.8 ± 0.9% CD38^+^ cells in *N* = 3 independent UCB donors; Figure S4). Traditional markers that would reflect the most naïve populations within HSPCs (CD90 and Lin) were also assessed via flow cytometry, but showed too little variance within the population of CD34^+^ cells from UCB for further analysis, as the majority of the cells were negative (population sizes of 97.8 ± 0.02% CD90^−^ and 99.6 ± 0.004% Lin^−^, respectively; Figure S5). In order to assess whether there is a correlation between the differentiation state of the cells and the presentation of shape/adhesive structures in the cells, the corrected total cell fluorescence (CTCF) was recorded after adhesion and immunofluorescent labelling of CD38, CD45RA and CD33. The surface levels of the present markers varied in the adherent fraction (Figure S6). Variation of these markers was also observed in the total CD34^+^ population via flow cytometry and transcriptomics (Figures S3 and S4), reflecting the various differentiation states present in the CD34^+^ cell populations. Therefore, marker abundance was categorized from *very low* to *very high* for CD38 and *negative* (−) to *high* for CD33 and CD45RA marker CTCF levels.

Next, we compared the frequencies of CD33 and CD38 marker levels of cells with the distinct analyzed cell shapes or adhesive structures. Most of the analyzed cells were CD38^low^CD33^low^ (Figure 4B–D), making up between 24% and 51% of cells in all categories. This indicated that most cells were naïve myeloid cells. However, when comparing round and elongated cells (Figure 4B), a larger fraction of round cells (17.8%) than of elongated cells (4.2%) was CD33^−^. Given that the production of CD33 indicates a commitment to the myeloid lineage, this suggested that myeloid progenitors tend to take on an elongated morphology. Furthermore, the population of cells that establish PLS a.t. did not contain any CD38^very high^ populations. Therefore, it appeared that cells producing PLS a.t. are less differentiated than cells that establish these structures b.t. (Figure 4C). Accordingly, CD38^very low^CD33^−^ and CD38^high^CD33^low^ populations were larger in cells with PLS a.t. (9.4% and 28.6% respectively) than in cells with PLS numbers b.t. (5.6% and 10.0%), indicating that these cells tend to be either not or early committed to the myeloid lineage (Figure 4C). Regarding filopodia establishment a.t., a tendency to show higher levels of CD33 and CD38 in comparison to cells with filopodia numbers b.t. was observed, indicating differentiation towards the myeloid lineage. This was supported by the finding that only <6% of cells with filopodia label were CD33^−^, compared to <20% of cells with filopodia establishment b.t. (Figure 4D). Regarding marker levels of CD45RA, it could also be observed that the populations of more naïve cells (CD38^very low^CD45RA^low^) were larger in round cells, cells with PLS a.t., and cells with filopodia b.t. than in the respective counterparts of these groups (Figure S7).

Statistically significant associations between the observed cell shape or characteristic adhesive structures establishment and marker level groups were detected via the Cochran‐Mantel‐Haenszel test (CMH‐test). Significant associations were detected between cell shape and CD33 levels, between filopodia establishment and CD33‐, CD38‐, and both marker levels combined, and between PLS establishment and CD38 and combined CD33‐/CD38‐marker‐levels. No significant correlations between the CD45RA‐marker‐levels and shape or PLS/filopodia establishment were detected (Table 2). Given that CD33 is a marker of myeloid differentiation and CD38 is used to discriminate between early and late CD34^+^ progenitor cells, the correlation between CD38 as well as CD33/CD38 levels and PLS establishment but not with cell shape (Table 2) is notable. This renders higher PLS levels an indicator of cell immaturity, given the lower CD38 levels in these cells. Cell shape (round vs. elongated), on the other hand, is correlated with CD33 levels only, indicating a connection to myeloid differentiation, with round cells being more likely non‐myeloid, indicated by higher numbers of CD33^−^ cells. The correlation between filopodia and CD38/CD33 levels, both individually and combined, shows that filopodia establishment is a strong indicator of myeloid differentiation in CD34^+^ progenitor cells, as levels of both markers are higher in these cells.

**TABLE 2:** *p*‐values for statistical associations between cell shape or structure establishment and differentiation state of HSPC. HSPCs were isolated from UCB and allowed to adhere to FN for 1 h. Cells were then labelled for the F‐actin cytoskeleton, determining their shape and structure category. Simultaneously, cells were IF‐labeled for the differentiation markers CD38, CD45RA, and CD33. CTCF values were acquired from IF microscopy to determine the marker levels. Marker level categories and shape/structure categories of each cell were then submitted to the Cochran‐Mantel‐Haenszel Test (CMH test) to test for statistical associations between marker level and cell category. Shown are the p‐values acquired via CMH‐test, significances are marked with asterisks (^*^≤ 0.05; ^**^≤ 0.01; ^***^≤ 0.001).

	Shape	Filopodia establishment	PLS establishment
CD33‐levels	0.02402^*^	0.002354^**^	0.9015
CD45RA‐levels	0.8602	0.1758	0.7946
CD38‐levels	0.1417	0.0001072^***^	0.005446^**^
CD33‐CD38‐combined‐levels	0.2156	0.002268^**^	0.03747^*^
CD45RA‐CD38‐combined‐levels	0.7245	0.1519	0.2465

As stem cell properties are associated with the cell‐cycle status, adherent cells were analyzed for the presence of Ki67, a marker well described to be only present in the nucleus during cell proliferation [27]. This analysis resulted in the finding that 100% of analyzed cells were undergoing proliferation (Ki67 CTCF values at least 100‐fold higher than respective signals in controls without primary antibody in *N* = 3 UCB donors; Figure S8).

Taken together, the CTCF values of progenitor markers in adherent HSPC fit the expression pattern of the respective genes in BM HSPC that showed a gradual increase across the population. The analysis of protein marker levels in the adherent cells indicated that cells that committed to myeloid progeny tend to take on an elongated morphology and to establish filopodia a.t. Vice versa, cells of round morphology and cells establishing PLS a.t. tend to be immature or early non‐committed progenitor cells (Figure 5).

**FIGURE 5:**
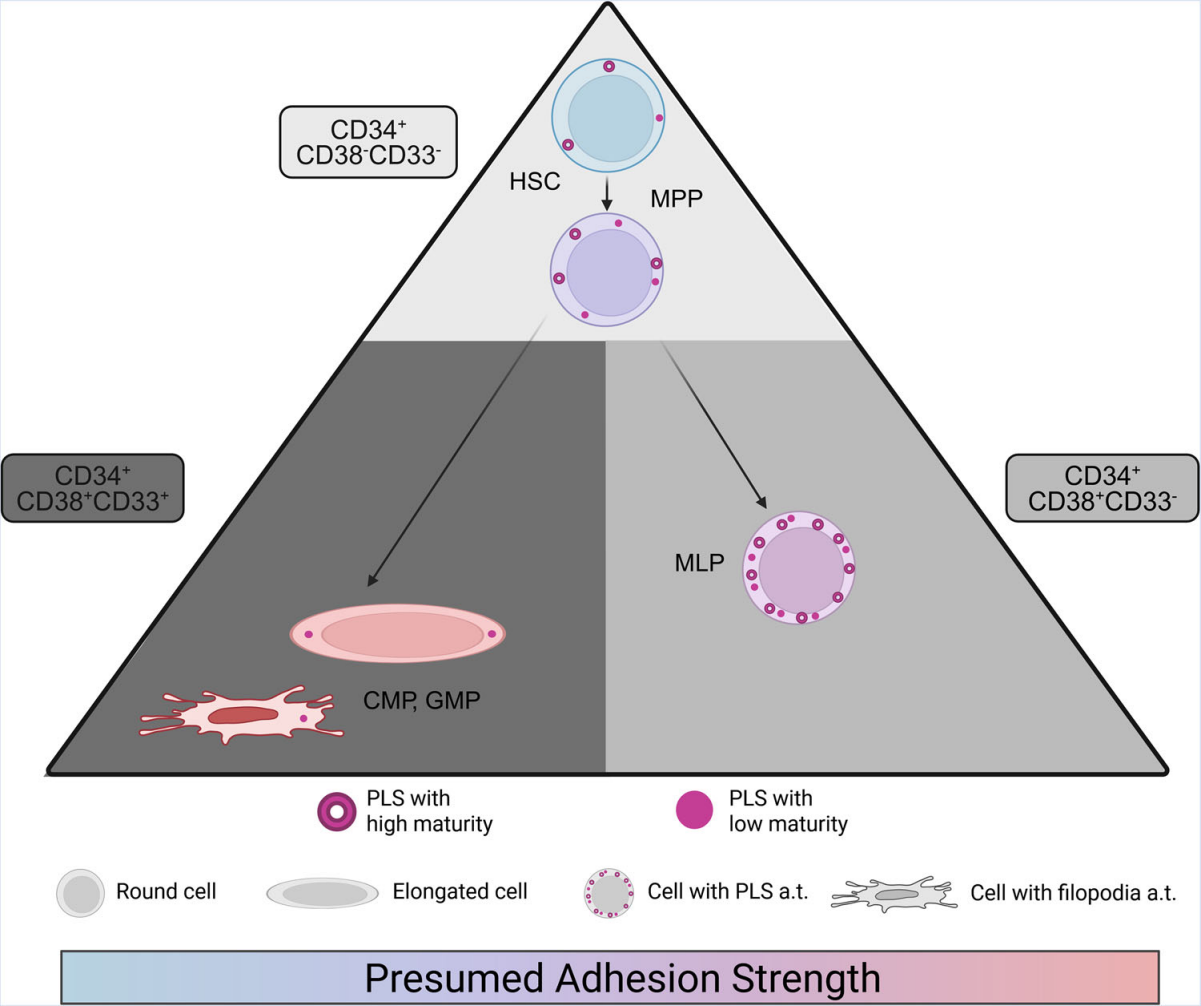
Overview of the association between HSPC differentiation state, morphology, and adhesive structures. Cells can be split into three groups according to marker production. Naïve CD34^+^ HSCs and MPP lack both CD38 and CD33 (light grey area of the triangle). Myeloid lineage commitment is indicated by additional CD33 expression (dark grey area). MLPs are CD34^+^CD38^+^CD33^−^ (intermediate grey area). Further differentiated cells and cells of myeloid lineage tend to take on an elongated cell shape and to establish filopodia a.t. Vice versa, round cells and cells with PLS establishment a.t. tend to be more naïve and/or are non‐myeloid, which might indicate commitment towards the lymphoid lineage. It is presumed that cells with an elongated cell shape and filopodia exhibit stronger adhesion forces. Round cells display higher numbers and more mature PLS than elongated cells or cells with filopodia. Therefore, it appears that in the absence of other adhesive structures, each PLS leads to rather low adhesion forces, and PLS number and maturity might be a measure for these cells to modulate their adhesion strength.

## 3 Discussion

In the here presented work the adhesion structures of HSPCs in contact with FN were analyzed. Initially, a heterogeneity in adhesion behavior, cell morphologies, and established adhesive structures was observed. This heterogeneity is likely the result of the highly dynamic, amoeboid‐like migration/attachment behavior that the cells exhibit. This style of migration was described before, as well as brought into context of various morphologies, for murine HSPC and the KG‐1a cell line, both cell types that closely resemble human HSPC [28]. Additionally, human HSPCs are able to attach to a substrate in vitro; however, they are not dependent on anchorage for survival [29, 30, 31, 32]. Furthermore, the CD34^+^ cell population from UCB is known to be quite heterogeneous, containing stem cells together with further differentiated progenitors, which contributes to the different cell morphologies and heterogeneous adhesion behavior [33, 34, 35, 36]. All these inherent properties of human HSPCs likely come together to serve the purpose of enabling the cells to react to a variety of signaling cues in vivo, making the dynamic adhesion behavior a feature of these cells [37, 38, 39]. It can be assumed that elongated cells or cells with many filopodia exhibit stronger adhesion forces than round cells or cells with high PLS establishment, respectively [40, 41, 42, 43, 44]. Interestingly, given the high prevalence of cells with round morphology or higher amounts of PLS within the presented study, this points to HSPCs adhering with low adhesion strength, which could serve the purpose of a quick response to extracellular signals. FN was chosen as a substrate due to its strongly adhesive properties, its ubiquitous presence throughout the ECM of the BM, and due to the fact that it is known for quiescence modulation in the context of HSPC integrins [6, 8, 9, 10, 11]. As FN can interact with a multitude of adhesion receptors, it is also able to regulate various cell functions through respective receptor interactions. These functions include homing, retention, proliferation, and migration of HSPCs specifically [6], which brings an additional variety to the interaction of the cells with their substrate.

The high prevalence of cells containing PLS in higher amounts is surprising, as the ability of HSPCs to establish these structures was largely unknown until it was described by Rademakers et al. [23]. In this study, the authors prompted the formation of PLS via stimulation of the cells with a phorbol ester, and the structures were put into the context of homing. Our work shows that adherent HSPCs display an inherent ability to form PLS in a highly dynamic manner. Therefore, PLS emerged as structures of interest for the present study.

The presence of pPYK2, vinculin, and paxillin within the observed F‐actin puncta in adherent HSPCs further confirmed the observed structures to be podosome‐like. However, the spatial distribution of the proteins of interest is surprising in parts, as the localization seems to be heterogeneously spread from the actin puncta core to its periphery, occasionally forming a ring structure. This distribution pattern of the proteins of interest could also be confirmed in the microscopy images, that not always aligned due to asymmetric assembly in the ring and the symmetric acquisition of the profiles. The asymmetric, geometrical assembly of the podosome ring is a known feature of the structures that was revealed with emerging super‐resolution microscopy techniques [45, 46]. In fully mature podosomes, the adhesive ring usually surrounds the F‐actin core [16], which differs from the heterogeneous display observed in HSPC here. The reason for this lies, most likely, in the highly dynamic turnover of these structures, which was speculated upon observing adhesion dynamics in live‐cell imaging. Fully mature podosomes are known to have a half‐life of 2–12 min [13], and the highly migratory behavior of some HSPCs, as discussed above, indicates a similar, if not higher, turnover of adhesion structures in these cells. Taken together, the data pointed to the formation of rather immature podosome structures that show highly dynamic assembly and disassembly, resulting in the heterogeneous display of structures even at the single‐cell level.

This prompted us to analyze whether significant differences in PLS architecture could be found on a larger scale between the various assigned groups of cell morphology and designated adhesive structures (PLS and filopodia) within the HSPC population. The FWHM analysis of the averaged RIP of each category revealed that PLS in HSPCs are considerably smaller than the core of fully matured podosomes. The FWHM of podosomes in, e.g., dendritic cells found in comparable studies is almost twice as large, at approximately 700 nm [47]. This is a further indication that PLS in HSPCs are an immature or atypical form of podosomes.

A higher FWHM in RIPs of podosomes usually indicates that the respective molecules are associated with the podosome ring rather than the core [47]. The significantly higher FWHM of the analyzed proteins of interest compared to the FWHM of F‐actin in cells of round morphology and round cells with PLS label indicates that the PLS established by these cells are more mature than in cells of the other assigned categories. Additionally, the difference between the FWHM values of proteins of interest as HSPCs take on an elongated cell shape and/or establish filopodia a.t. becomes less significant, which reflects back on the adhesion strength of these cells, as mentioned above. Together with the finding that cells not displaying elongation or filopodia a.t. showed PLS higher in number and/or maturity, it appears that PLS maturity and number might be parameters to modulate adhesive forces (Figure 5).

Lastly, we investigated whether a correlation between cytoskeletal presentation and differentiation status of HSPC exists. In analyzing the CTCF values of progenitor markers in adherent HSPCs, the majority of the adherent HSPCs were positive for the assessed markers, which, in reference to the transcriptomic data, puts them on the trajectory of early committed myeloid cells, independent of cytoskeletal presentation. This finding fits reports that the CD34^+^ cell population isolated from UCB tends to be enriched with cells of myeloid progeny [48]. Additionally, all analyzed cells were undergoing proliferation. One explanation for both findings might be the overnight culture of the cells in HPC expansion media containing thrombopoietin, stem cell factor, Fms‐like tyrosine 3 ligand, and interleukin 3, cytokines that are well established to modulate HSPC self‐renewal and expansion and favor proliferation and myeloid differentiation in concert with each other [49, 50]. Thus, the analyzed adherent cell fraction was a heterogeneous, actively cycling HSPC population with a myeloid bias. Evaluating the progenitor marker levels of adherent HSPCs in concert with cytoskeletal presentation, we found that further differentiated cells, especially those further committed to myeloid lineage, tend to take on an elongated cell shape and establish filopodia a.t. This correlation fits with the notion that HSPCs differentiate when they exit their niche via migration, which is accompanied by an elongated cell shape [38]. The tendency for myeloid cells to establish filopodia in response to immunomodulation is also well documented [51, 52]. More interestingly, given the aim of this research to investigate adhesion structures of HSPCs in the context of retention, there is an indication of a correlation between less differentiated cells and enhanced PLS establishment. We also find that round cells and cells with PLS a.t. show a lack of CD33 and might therefore be primed for differentiation into the lymphoid lineage; however, the statistical analysis regarding CD45RA correlation did not give a significant result. Taken together, our findings indicate that less committed cells, closest to the population with stem‐cell properties, might establish PLS to remain in the BM niche.

Within the work presented here, we report the establishment of PLS by HSPCs. We show that these structures are more mature in round cells and in round cells with PLS number a.t. Cells displaying these characteristics are also the majority among the multitude of cytoskeletal morphologies observed. We find strong indications that these round and PLS‐rich HSPCs are also less differentiated. Given the associations we find between PLS establishment, PLS maturity, and cell differentiation state, we come to the conclusion that PLS are modulating adhesion forces in naïve HSPCs to retain them in their niche. Interestingly, the findings open up new perspectives on calcium‐dependent regulation of HSPCs. As recent reports describe a long term‐HSC population marked by high Ca^2+^‐influx and high intracellular Ca^2+^‐concentration, and given the prominent role of pPYK2, whose autophosphorylation is influenced by calcium influx [53, 54, 55], in the here‐discussed structures, the PLS might play a crucial role in HSC maintenance. Given that this particular population of interest is present under hypoxic conditions, as they are present in the endosteum of the BM, the role of PLS might also be further evaluated in relation to the ECM protein osteopontin, which makes up around 2% of the non‐collagenous protein in the endosteum and has been shown to specifically promote quiescence in HSPCs [56, 57]. In conclusion, the adhesion structures of HSPCs outlined in this study present an intriguing area for further exploration into the mechanisms that are involved in the interaction between HSPCs and the hematopoietic niche. Gaining insight into the interactions within the adhesion machinery of HSPC as well as the interactions between cells and the ECM is a crucial step in differentiating the mechanisms of retention, homing, mobilisation and migration within the HSC niche.

## 4 Methods

### 4.1 Isolation and Maintenance of HSPCs from human UCB

CD34^+^ cells were isolated from human UCB, obtained after informed consent of the mothers at the Gynecological Hospital of Hannover Medical School. Approval of the local ethics commission was obtained and maintained throughout the study (EV LUH 06/2019). Once UCB was collected after birth, heparin was added, and the sample was kept on a shaking device at RT until isolation of cells was performed to prevent clotting. Isolation of HSPC was performed 24–48 h after UCB collection. First, the blood was submitted to density gradient centrifugation using the Pancoll human separation media (Density: 1.077 g ml^−1^, PANBiotech, Aidenbach, Germany). The fraction containing mononuclear cells was then submitted to magnetic affinity cell sorting using the CD34 MicroBead Kit human (Milteny‐Biotech, Bergisch‐Gladbach, Germany) according to the manufacturer's instructions. Isolation of CD34^+^ cells was verified via Flow Cytometry using an anti‐CD34‐FITC antibody (supplier ID: 130‐113‐740; diluted 1:50; Milteny‐Biotech, Bergisch‐Gladbach, Germany). Only if >95% of cells were positive for CD34, they were submitted to further analysis. Cells were cultured in Hematopoietic Progenitor Expansion Medium XF with Cytokine Mix E for 24 h before freezing in Freezing Medium Cryo‐SFM (all Promocell, Heidelberg, Germany). Cells were again cultured for 24 h after thawing before further analysis.

### 4.2 Adhesion of HSPC to Fibronectin

Wells of a removable 8‐well cell culture chamber on a glass slide (SARSTEDT, Nümbrecht, Germany) were coated with 20 µg mL^−1^ fibronectin. To each coated well 5 × 10^4^ cells were added in adhesion medium containing 25 µm MnCl_2_ in Roswell Park Memorial Institute (RPMI) 1640 medium (Merck, Darmstadt, Germany) and left to adhere for 1 h at 37°C and 5% CO_2_. Nonadherent cells were then removed by washing the wells once with adhesion medium. Adherent cells were submitted to microscopic analysis.

### 4.3 Immunofluorescent Staining

Adherent HSPCs were fixed with 4% paraformaldehyde for 15 min at room temperature (RT). After washing with PBS (containing MgCl_2_ and CaCl_2_) cells were permeabilized with 0.1% Triton X‐100 for 5 min at RT and washed again. To block nonspecific binding sites, cells were incubated with 1% Bovine Serum Albumin (BSA) for 1 h at RT. After washing, the cells were incubated with primary antibody in 0.1% BSA solution for either 1 h at RT or 4°C overnight (o/N), depending on the antibodies used.

Primary antibodies used for immunofluorescent staining in this study were the anti‐human CD38 (AB_2789947; polyclonal; used at 300 µg mL^−1^, incubated o/N), anti‐human Phospho‐Pyk2 (Tyr402; AB_2533697; polyclonal, used at 800 ng mL^−1^, incubated o/N), anti‐human vinculin (clone: VLN01; AB_10976821; used at 4 mg mL^−1^, incubated 1 h), recombinant Ki‐67 (clone: SP6, MA5‐14520; monoclonal, used at 124 ng mL^−1^, incubated o/N) (all Invitrogen by Thermo Fisher Scientific, Waltham, MA USA), anti‐human paxillin (clone: 349/Paxillin; AB_397464; used at 2.5 µg mL^−1^, incubated o/N; BD, Kelberg, Germany) and anti‐human CD33 (clone:WM53; ab30371; used at 5 µg mL^−1^, incubated o/N; Abcam, Cambridge, UK) antibodies.

For negative controls, primary antibodies were omitted. After washing all samples were incubated with secondary antibodies (Alexa Fluor 647‐conjugated goat anti‐mouse (AB_2535805) and Alexa Fluor 568‐conjugated goat anti‐rabbit (AB_10563566), 2 µg mL^−1^; both Invitrogen by Thermo Fisher Scientific, Waltham, MA USA) together with Alexa Fluor 488‐conjugated Phalloidin (ab176753; Abcam, Cambridge, UK) to label F‐actin in 0.1% BSA for 30 min at RT in the dark. After final washing, the samples were mounted on a high‐precision coverslip (Roth, Karlsruhe, Germany) with Mowiol mounting medium.

### 4.4 Confocal Laser Scanning Microscopy (cLSM)

Immunofluorescently labeled cells were imaged using a Zeiss LSM 980 Airyscan system with an Airyscan detector. All confocal images show a single confocal layer with the ventral cell side in focus. For time‐series imaging, cells were kept at 37°C and 5% CO_2_ in the imaging system, and images were taken at the same location for 1 h with a 10 min interval.

### 4.5 Flow Cytometry

HSPC were stained with CD33‐PE (AB_314347; diluted 1:20; BioLegend, San Diego, CA USA), CD34‐FITC (supplier ID 130‐113‐740), CD38‐APC (supplier ID 130‐124‐044) antibodies (both diluted 1:50; Milteny‐Biotech, Bergisch‐Gladbach, Germany) and respective isotype controls for 10 min in 4°C in PBS containing 1% EDTA and 0.5% BSA. Analysis was then performed using the FACSVerse Flow Cytometer, and data processing utilized the FlowJo v.10.9.0 software (BD, Kelberg, Germany). For spectral flow cytometer HSPC were stained with CD34‐FITC (supplier ID #343503), CD33‐BV421 (supplier ID #366621), CD45RA‐APC‐Fire810 (supplier ID #304191), CD90‐BV650 (supplier ID #328143), Lin‐PacificBlue (antibody cocktail containing CD3, CD14, CD16, CD19, CD20, CD56 antibodies, supplier ID #348805; all diluted 1:40) and CD38‐BV785 (Supplier ID #303529, diluted 1:20; all BioLegend, San Diego, CA USA) for 10 min at 4°C in PBS containing 1% EDTA and 0.5% BSA. Compensation beads (supplier ID #424601, BioLegend, San Diego, CA, USA) were labelled with all antibodies mentioned above individually. Analysis was then performed using the Northern Lights spectral flow cytometer from Cytek (Fremont, CA, USA), and data processing utilized the FlowJo v.10.9.0 software (BD, Kelberg, Germany).

### 4.6 Image and Data Analysis

Images obtained by cLSM were processed using the free software ImageJ [58] with the Plug‐ins “Poji” [59] and “FiloQuant” [60].

Radial intensity profiles of PLS were obtained utilizing the “Poji” plug‐in. Radial intensity profiles were submitted to gaussian fit using the software Origin (9.9.0.220; OriginLab Corporation, MA, USA). Full width at half maximum (FWHM) of fitted profiles with R^2^ ≥ 95% were submitted to One‐way ANOVA with a Tukey post hoc test to assess statistical differences.

CTCF values were acquired by subtracting the product of the cell/nucleus area *A*_cell/nucleus_ and the mean fluorescence intensity of the background readings MFI_Background_ from the integrated density of the marker reading of the cell IntDen_Marker_ Equation (1).

(1) $$\mathrm{CTCF}={\mathrm{IntDen}}_{\mathrm{Marker}}\;-\;\left(A_{\mathrm{cell}/\mathrm{nucleus}}\;\times \;{\mathrm{MFI}}_{\mathrm{Background}}\right)$$

CTCF values of the markers CD38, CD33, and CD45RA were categorized from *very low* to *very high* (CD38) and *negative* (−) to *high* (CD33 and CD45RA), utilizing the thresholds reported in Table S1.

Statistical association of CD‐marker levels according to CTCF and morphology was analyzed via the Cochran‐Mantel‐Haenszel test, accounting for the *N* = 3 UCB donors as strata.

### 4.7 Morphology Assessment

The morphology of each adherent HSPC from UCB was assessed in order to get a better understanding of the established adhesion structures in the respective morphology group. Each cell was assigned either an arc, round, or elongated morphology. Arc morphology cells were defined by cell area. Cells not meeting the critical value for area were assigned either round or elongated morphology according to aspect ratio (AR). Additionally, in these assessments regarding cell shape, it was determined whether cells showed filopodia or PLS presenting morphologies. To this end, cells were defined as having filopodia morphology if the filopodia number per cell and the mean filopodia length, as detected by the plug‐in FiloQuant, exceeded the threshold value. Both these criteria needed to be met to exclude cells with many short or few long filopodia. Analogously, cells were considered to display PLS morphology if the number of PLS detected by the plug‐in “Poji” and/or the PLS density exceeded the threshold value. Threshold values are listed in Table 1. Threshold values regarding cell area, filopodia number, mean filopodia length, PLS number, and PLS density are the median of these values between cells strongly displaying the respective morphology and the rest of the total cell population.

### 4.8 Single Cell HSPC Data

HSPC raw data from Pellin et al. [24] were downloaded via GEO. Using Python (v3.11.13; Python Software Foundation, OR, USA) and Scanpy (v1.11.4; [61]), the .tsv matrix was converted into an AnnData (v0.12.0) object. Cell type names were assigned based on filenames, and mitochondrial (MT‐), ribosomal small subunit (RPS‐), ribosomal large subunit (RPL‐), and hemoglobin (HB) genes were annotated for quality control. Scanpy's “calculate_qc_metrics” function was used to compute QC metrics for downstream filtering. Outliers were identified using an asymmetric median absolute deviation (MAD) approach, calculating separate variability measures below and above the median to better capture the skewed distribution typical of gene expression data. Cells were flagged as outliers if their metric values exceeded ±3 MAD from the mean, using the respective asymmetric MAD. This threshold was applied as an upper bound only for mitochondrial content, and as two‐sided bounds for total counts, gene counts, and the percentage of counts in the top 20 expressed genes. Cells exceeding these thresholds were removed from the dataset (8.91% of cells). Doublet detection was performed using both Scrublet (v0.2.3; [62]) and SCVI's (v1.3.2; [63]) SOLO algorithms. Scrublet was applied to each sample individually, labeling the top 5% of doublet scores as doublets. Samples were then merged, and SOLO was run on the combined dataset with a probability threshold of 0.98. Doublet calls from both methods were combined, and all identified doublets were excluded (4.7% of cells). The dataset was then subset to the desired cell types, and raw counts were stored in an additional AnnData layer named counts. Counts were normalized to a target sum of 10,000 using Scanpy's “normalize_total” function and log‐transformed with “log1p”. A nearest neighbor graph was constructed with “neighbors” and embedded into two dimensions using UMAP (minimum distance = 0.001). For differential expression analysis, raw count data from each sample were aggregated into three pseudobulk replicates by randomly partitioning cells into equal groups and summing their counts per gene. Pseudobulk matrices were constructed in Scanpy and exported, along with replicate metadata, to R via rpy2 (v3.6.1). DESeq2 (v1.48.1; [64]) was used to fit Wald tests comparing each sample to all others combined, with cell type specified as a categorical variable in the design formula. Significance was defined as adjusted 𝑝 < 0.05 for each comparison.

## Author Contributions

**Miriam Greve**: conceptualization, investigation, Writing – original draft, methodology, validation, visualization, Writing – review and editing, formal analysis, resources, data curation. **Peter Schertl**: supervision, resources, project administration, writing – review and editing, methodology, conceptualization. **Frank Schaarschmidt**: formal analysis, writing – review and editing. **Constantin von Kaisenberg**: resources, writing – review and editing. **Cornelius Miller**: software, visualization, writing – review and editing, writing – original draft. **Susanna Spindler**: investigation, visualization. **Sophia Rudorf**: software, writing – review and editing. **Cornelia Lee‐thedieck**: conceptualization, funding acquisition, writing – review and editing, methodology, project administration, supervision, resources.

## Funding

This work was supported by the Ministry of Science and Culture (MWK) of Lower Saxony, Germany, through the SMART BIOTECS alliance between the Technical University Braunschweig and the Leibniz University Hannover. The project was also supported as part of the collaborative research project Matrix Evolution, funded by zukunft.Niedersachsen, a funding programme of the Lower Saxony Ministry of Science and Culture (MWK) and the Volkswagen Foundation.

## Figure Preparation

Schemata included in figures were created in BioRender. Schertl, P. (2026) (https://biorender.com/k1vac43) and using the Servier Medical Art, provided by Servier, licensed under a Creative Commons Attribution 3.0 Unported license.

## Conflicts of Interest

The authors declare no conflict of interest.

## Supporting information




**Supporting File**: adbi70159‐sup‐0001‐SuppMat.pdf.

## Data Availability

The data that support the findings of this study are available from the corresponding author upon reasonable request.
