# Supplementary material for: Hematopoietic Stem and Progenitor Cells Establish Dynamic Podosome‐Like Structures to Interact with Fibronectin
Source: Adv Biol (Weinh). 2026 Sep 27;10(9):e70159. doi: 10.1002/adbi.70159 (PMC13617278; doi:10.1002/adbi.70159)
Supplement: Supplementary file 1 [file ADBI-10-e70159-s001.pdf]

# Supplementary Data

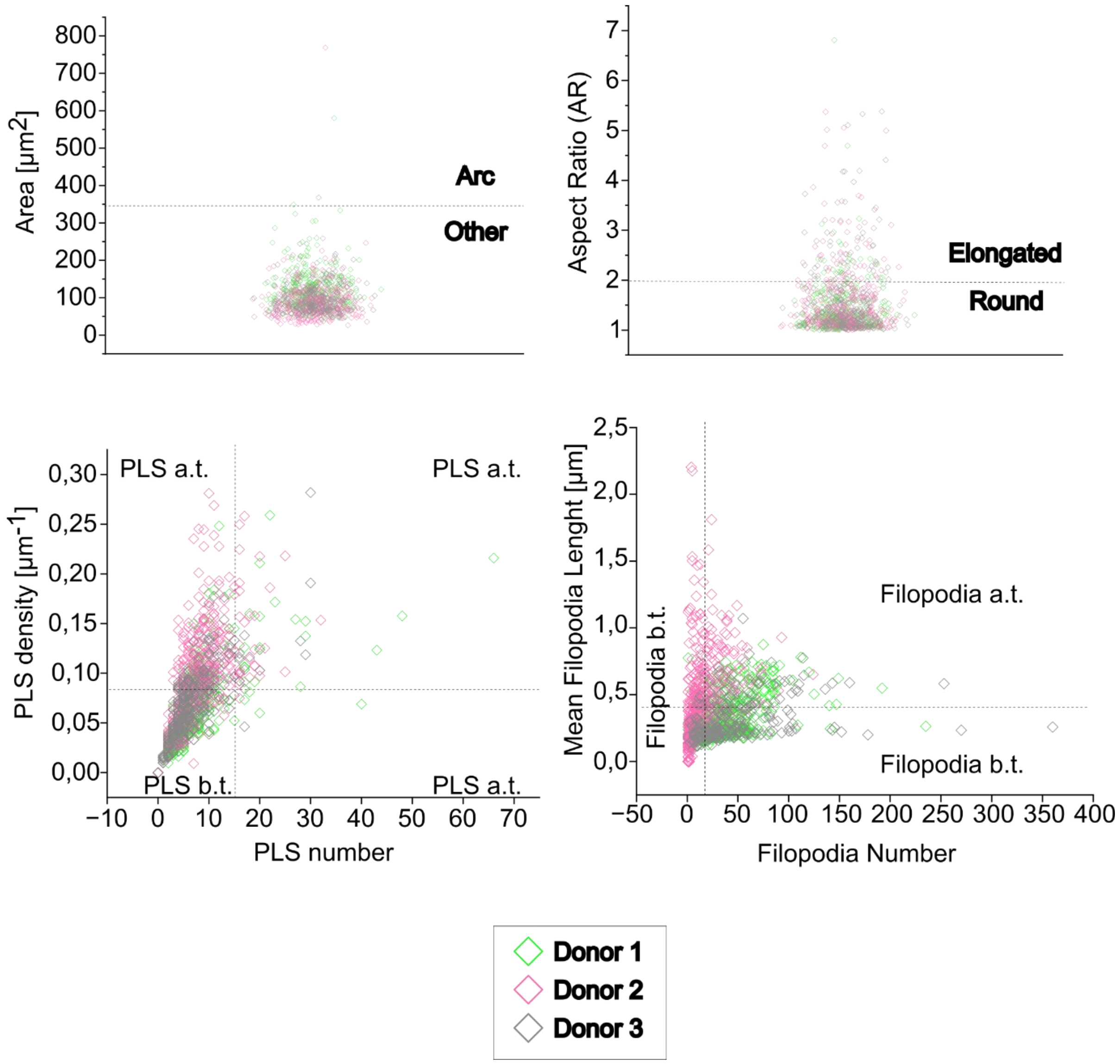


**Figure S.1: Distribution of parameters for morphology determination** Shown is the distribution of values determining cytoskeletal description acquired after adhesion of HSPC to FN for 1 h and subsequent cLSM imaging. N=3 independent cord blood donors are labelled by color and threshold values are indicated by doted lines, alongside the respective label determined by this threshold.

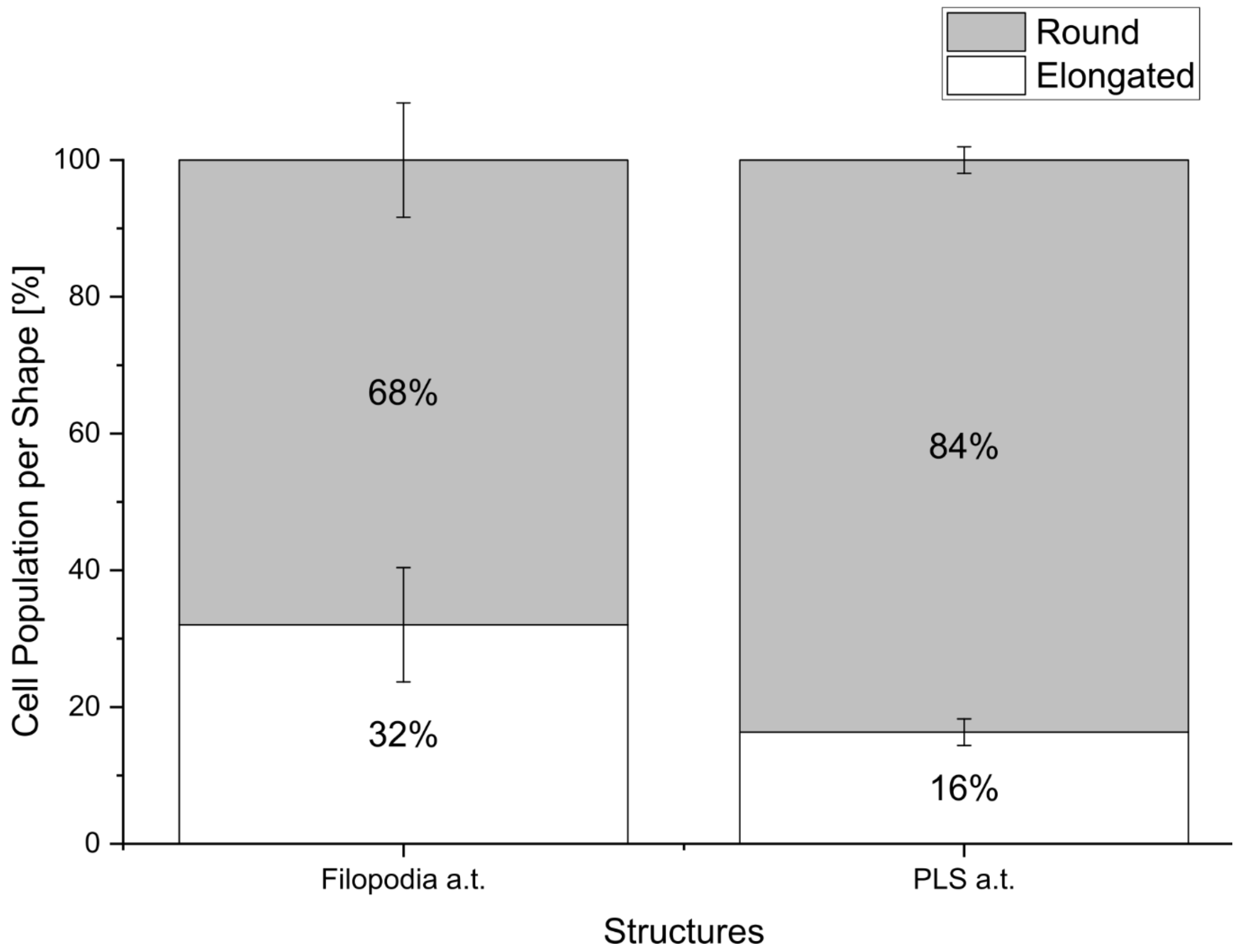


**Figure S.2: Percentage of round or elongated HSPC respective to filopodia or PLS abundance.** HSPC were isolated from UCB and let to adhere to FN for 1 h. While 68% of cells with filopodia above threshold (a.t.) show an elongated morphology, 84% of cells that establish PLS a.t. are of round cell shape. Depicted are the mean (± SD) population sizes of respective categories of N=3 independent UCB donors.

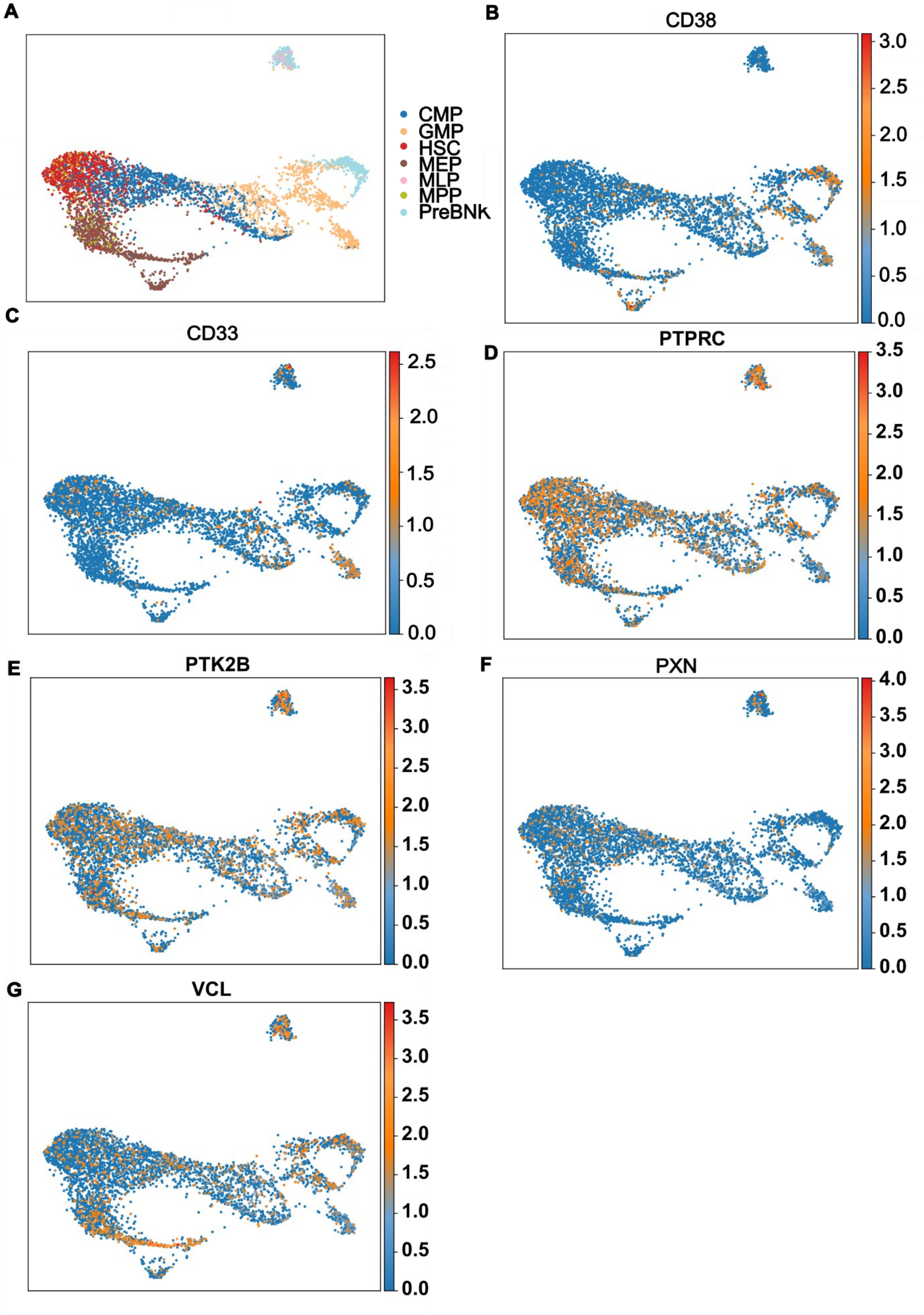


**Figure S.3: Expression of progenitor and adhesion markers are evenly distributed across the BM HSPC population.** Single cell transciptomics data of BM HSPC from Pellin et al. (2019) was submitted to nearest neighbor plotting. (A) Annotations of HSPC populations from source data were kept and are labelled by color,

revealing the shifting progeny populations. CMP: common myeloid progenitors; GMP: granulocyte-monocyte progenitors; HSC: hematopoietic stem cells; MEP: megakaryocyte-erythroid progenitors; MLP: multi-lymphoid progenitors; MPP: multi-potent progenitors; PreBNK: Pre B- and natural killer cells; (B-F) Relative gene expression of (B) CD38, (C) CD33, (D) PTPRC, (E) PTK2B, (F) PXN and (G) VCL in BM HSPC in nearest neighbor plot. Color scale indicates the relative expression level of the respective gene in every single cell showing the shift of gene expression along the path of differentiated progenitors as annotated in (A).

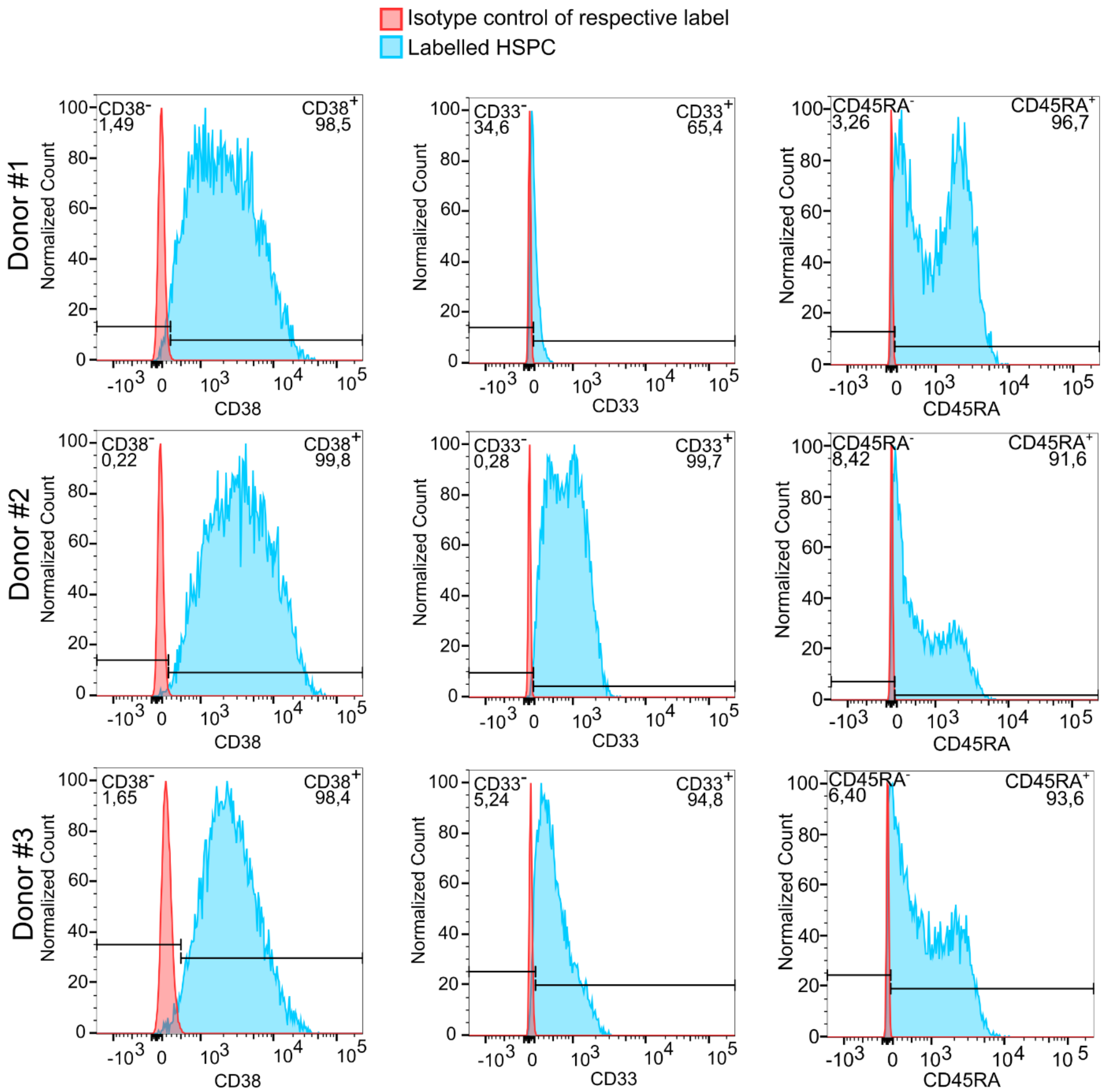


**Figure S.4: Percentage of CD38-, CD45RA and CD33-positive and negative cells per UCB donor.** Cells were submitted to Flow Cytometry after isolation $CD34^+$ cells from UCB and labelling them for CD38, CD33 and CD45RA. Depicted are the histograms for CD38- (left column), CD33- (middle column) and CD45RA (right column) labelled cell counts of N=3 independet UCB donors (from top to bottom). Cells stained with isotype antibody as control are depicted in red and determine the 1 percentile of positive cells, effectively limiting false negatives to 1%. The percentage of positive and negative cells are indicated in the corners of the histograms respectively.

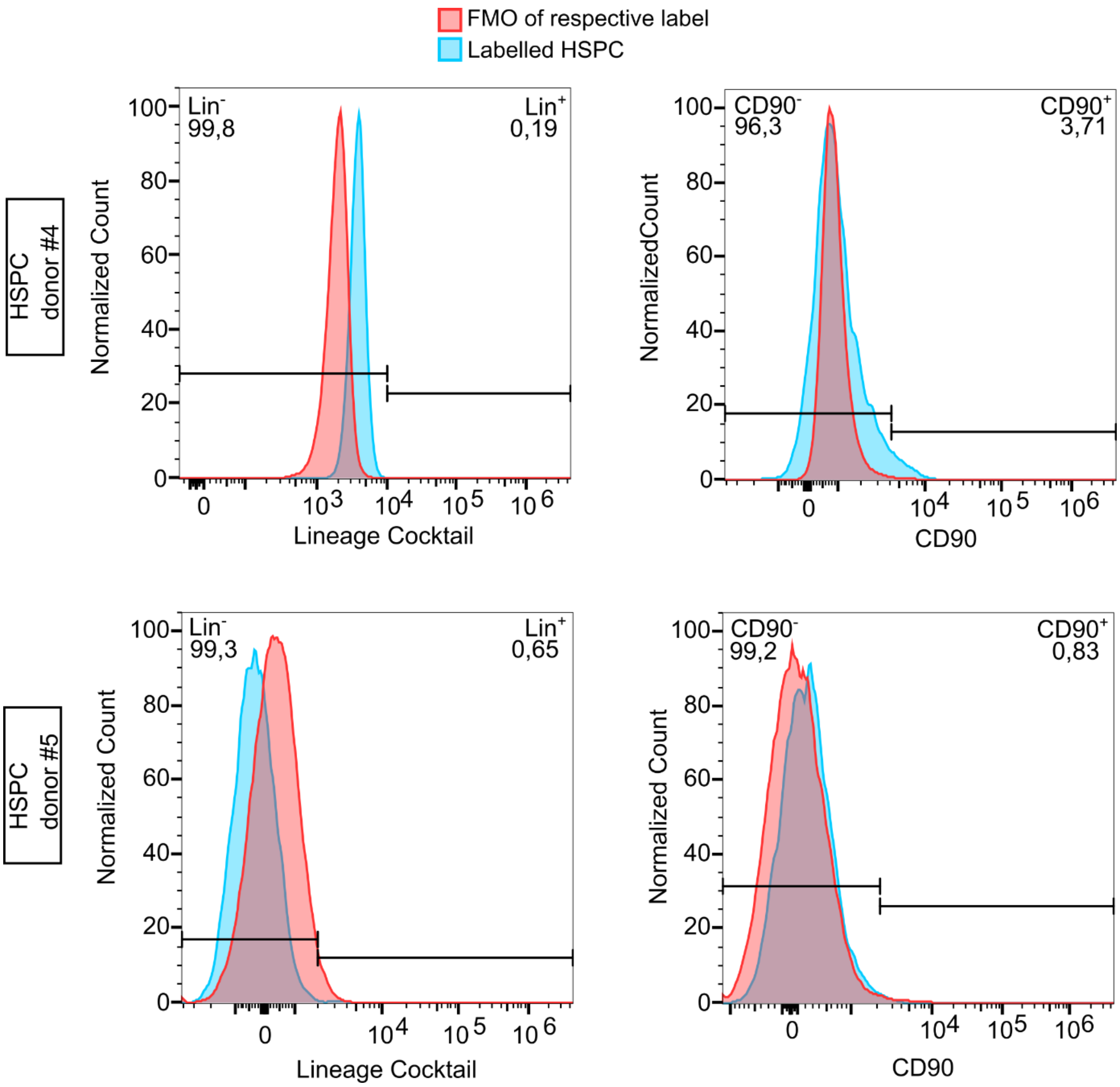


**Figure S.5 Percentage of Lin- and CD90-positive and negative cells per UCB donor.** Cells were submitted to Flow Cytometry after isolation CD34$^+$ cells from UCB and labelling them for Lineage Cocktail and CD90. Depicted are the histograms for Lineage- (left column) and CD90 (right column) labelled cell counts of N=2 independent UCB donors (from top to bottom). Cells stained with fluophores-minus-one (FMO) as control are depicted in red and determine the 1 percentile of positive cells, effectively limiting false negatives to 1%. The percentage of positive and negative cells are indicated in the corners of the histograms respectively.

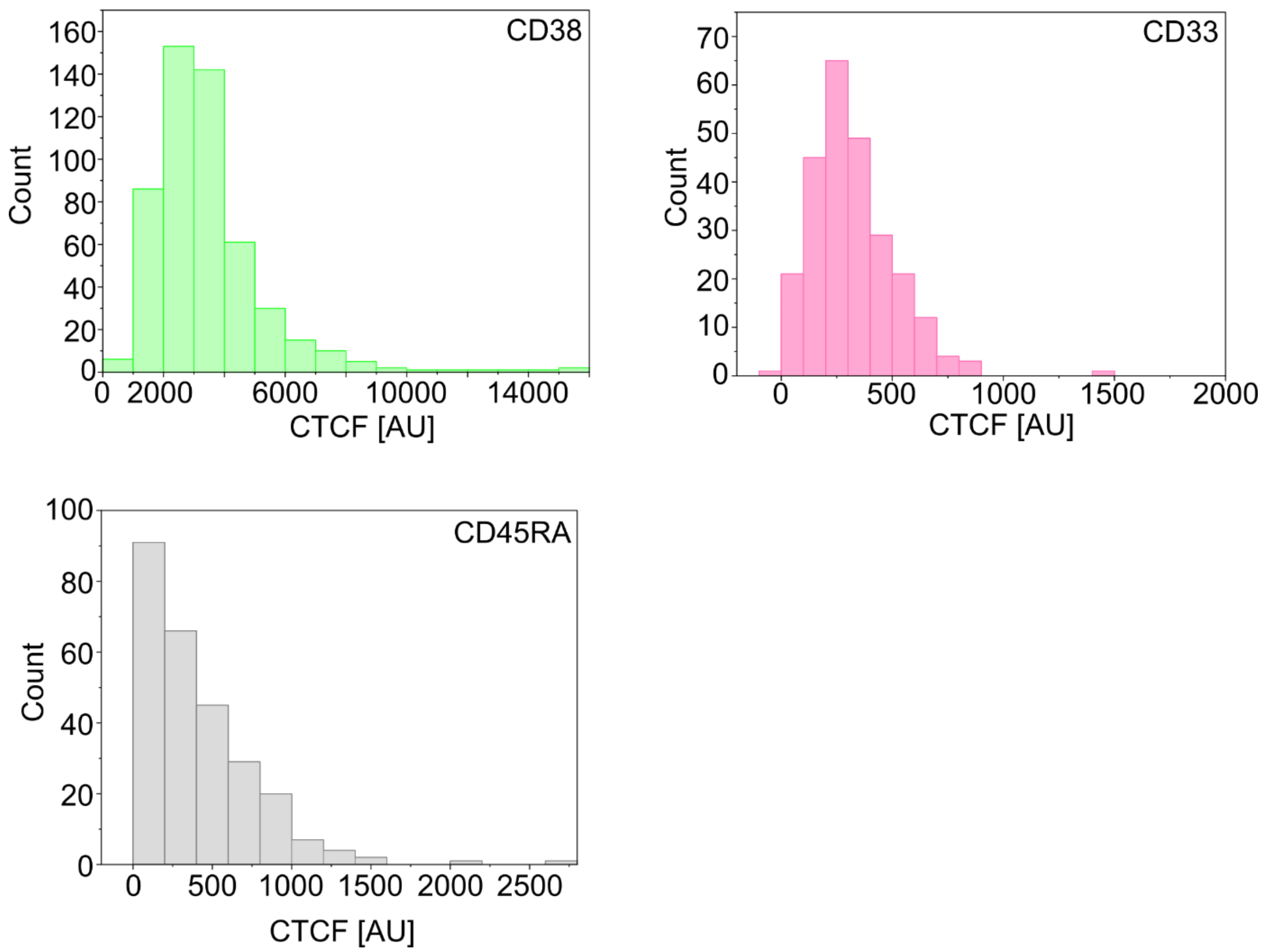


**Figure S.6: Histograms of CD38, CD33 and CD45RA CTCF values.** After isolation from UCB cells were let to adhere to FN for 1 h and immunofluorescently labelled for CD38, CD33 and CD45RA. CTCF values of respective labelings were acquired for each cell. Depicted are histograms of CTCF values for the total analyzed cell population from N=3 independent UCB donors.

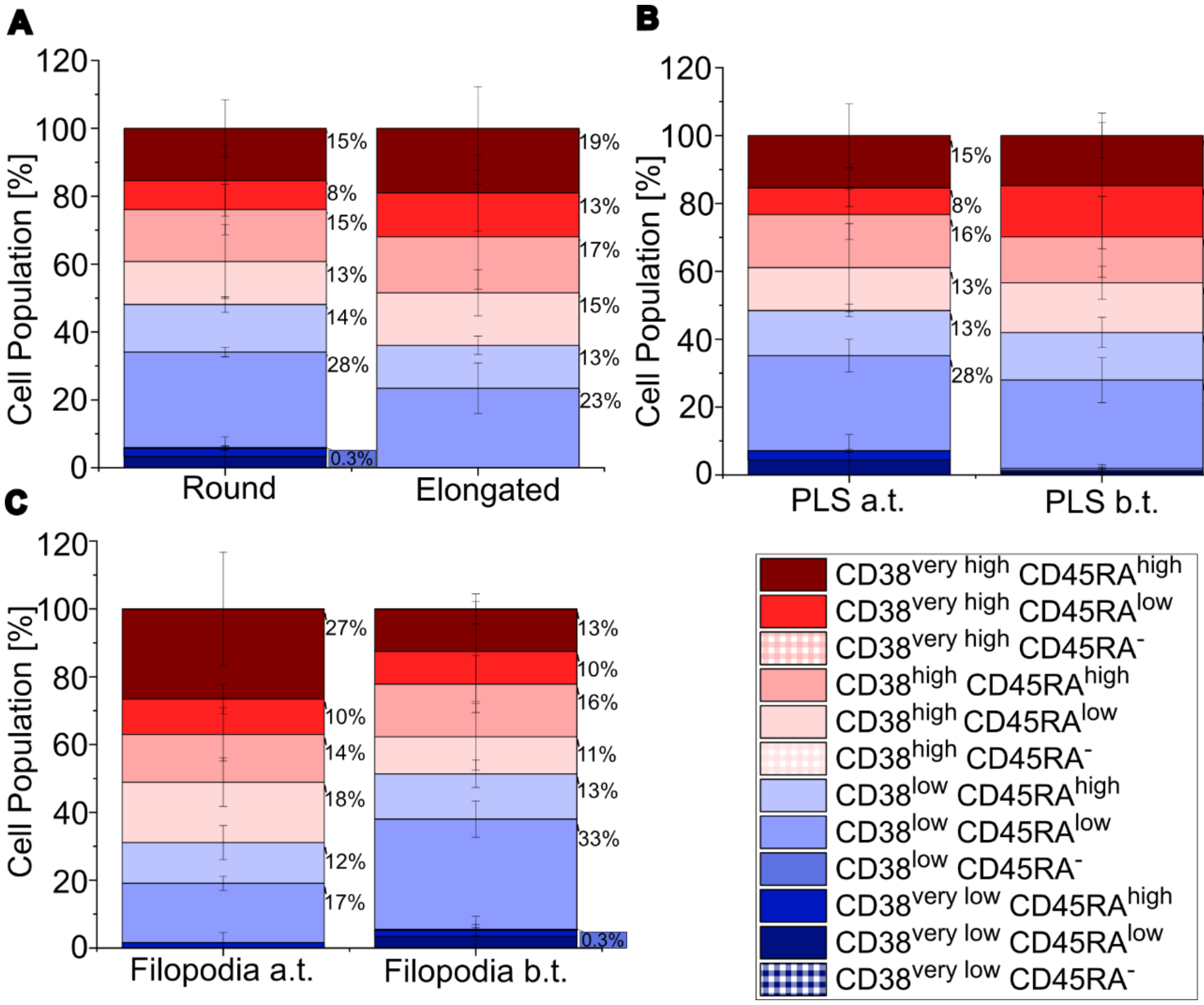


**Figure S.7 Distribution of CD45RA abundance in HSPC of different shape and establishment of cytoskeletal structures** (A-C) HSPC were isolated from UCB and assessed for progenitor marker production of CD38 and CD45RA via Flow Cytometry prior to adhesion. Marker production was assessed via IF microscopy after adhesion to FN for 1 h, and CTCF of the labelled markers were determined. Progenitor marker production levels of cells were grouped according to CTCF values, by transferring population sizes observed in flow cytometry to adhered cells via median CTCF and rule of three. Stacked column graph representing the population sizes of respective marker levels in percent (±SD) of n=265 individual cells from N=3 independent UCB donors. Shown are the population sizes for adherent HSPC with (A) round vs. elongated morphology, (B) PLS a.t. vs. PLS b.t., (C) filopodia a.t. vs. filopodia b.t.reshold, respectively. Mean Percentages > 5% are shown next to the respective bar segments. CD38low

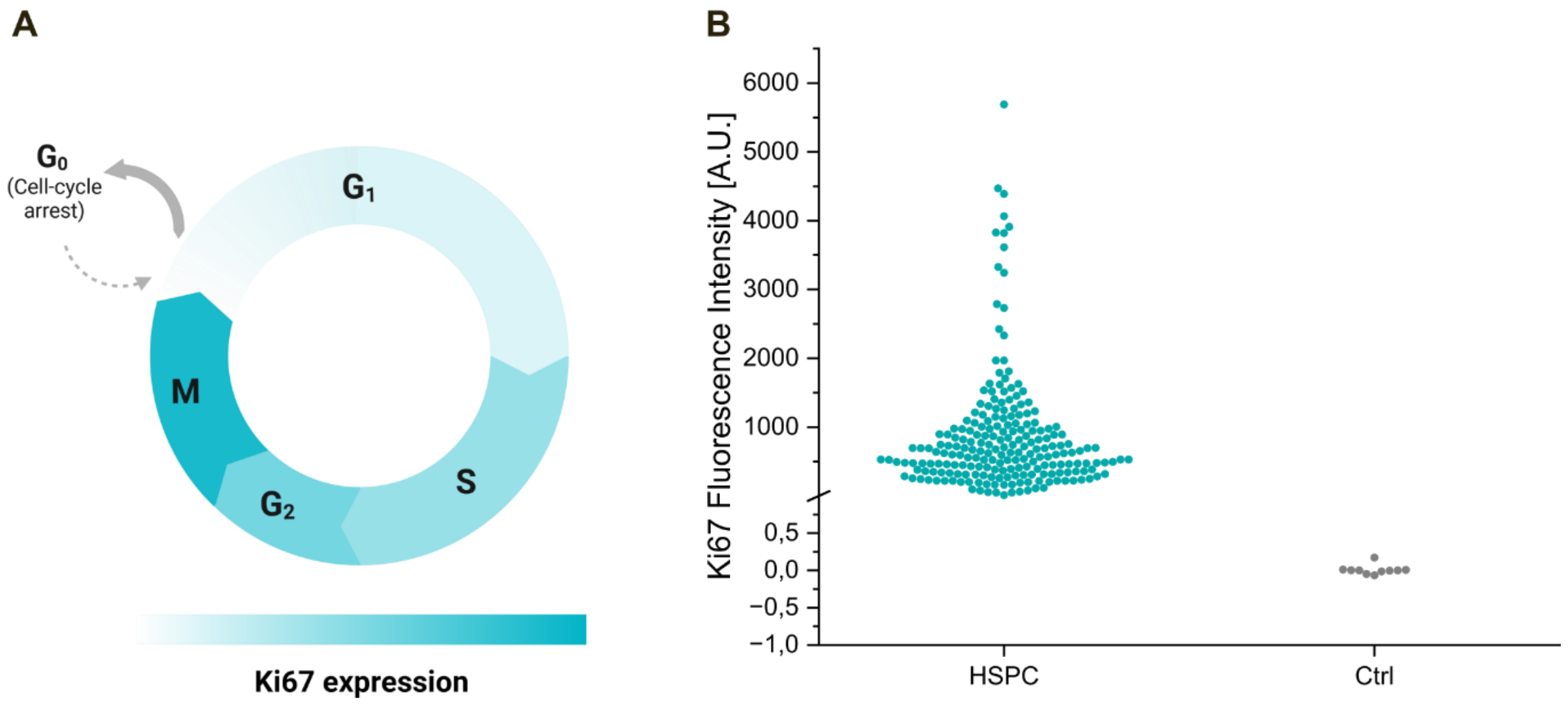


**Figure S.8: Ki67 intensity level in adherent HSPC** (A) Schematic depicition of Ki67 expression during cell cycle progression. Ki67 expression rises continuously from G1- to S- to G2- through to M-phase. Ki67 is absent from the nucleus if a cell exists the cell cycle and becomes quiescent. (B) Ki67 fluorescence intensity within the nucleus of adherent HSPC. Shown are the CTCF values for Ki67 of all adherent HSPC alongside the CTCF values of a control (ctrl) were primary Ki67-antibody was omitted. As CTCF values for all adherent HSPC are at least 100-fold higher than the values of respective controls, 100% of cells are considerer to undergo proliferation.

**Table S.1: CTCF threshold values for assignment of marker descriptors**. After isolation from UCB cells were let to adhere to FN for 1 h and immunofluorescently labelled for CD38, CD33 and CD45RA. CTCF values of respective labelings were acquired for each cell.

| Marker descriptor | CTCF threshold value |
|---|---|
| CD38$^{-}$ | <65 |
| CD38$^{\text{very low}}$ | <1620 |
| CD38$^{\text{low}}$ | <3240 |
| CD38$^{\text{high}}$ | <4860 |
| CD38$^{\text{very high}}$ | >4860 |
| CD33$^{-}$ | <150 |
| CD33$^{\text{low}}$ | <440 |
| CD33$^{\text{high}}$ | >440 |
| CD45RA$^{-}$ | <35 |
| CD45RA$^{\text{low}}$ | <320 |
| CD45RA$^{\text{high}}$ | >320 |
